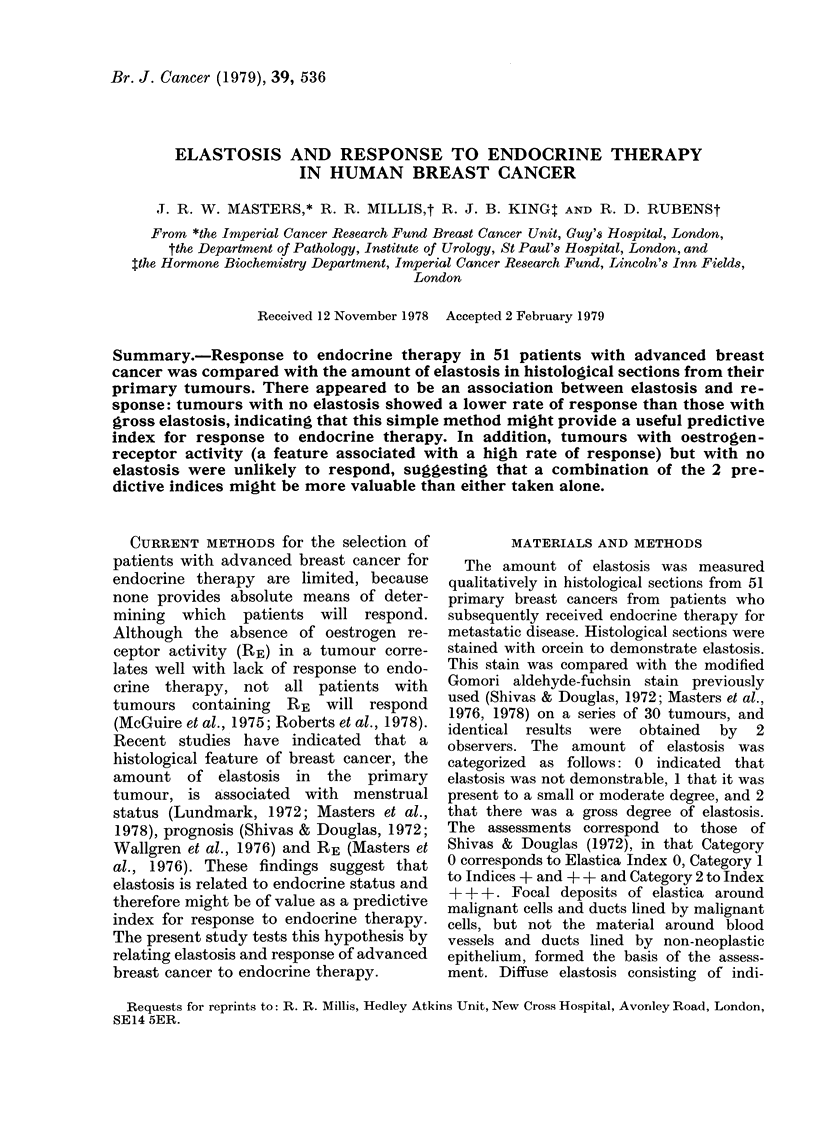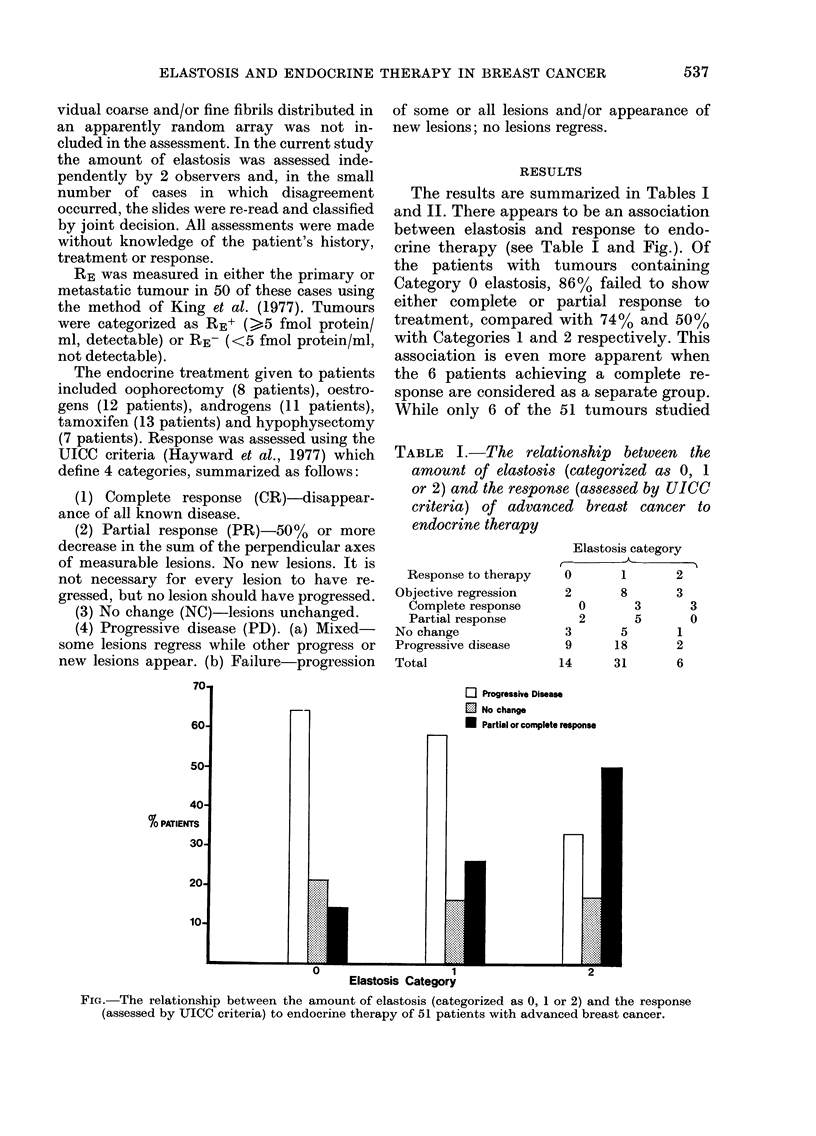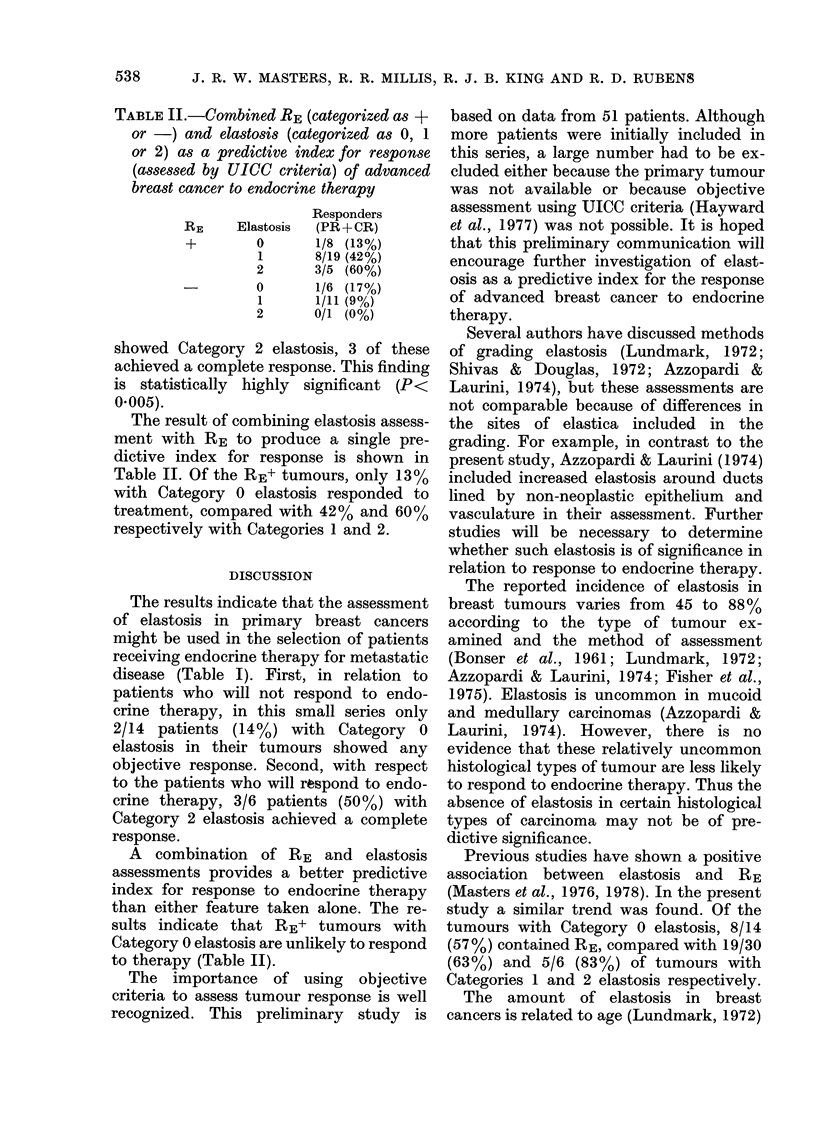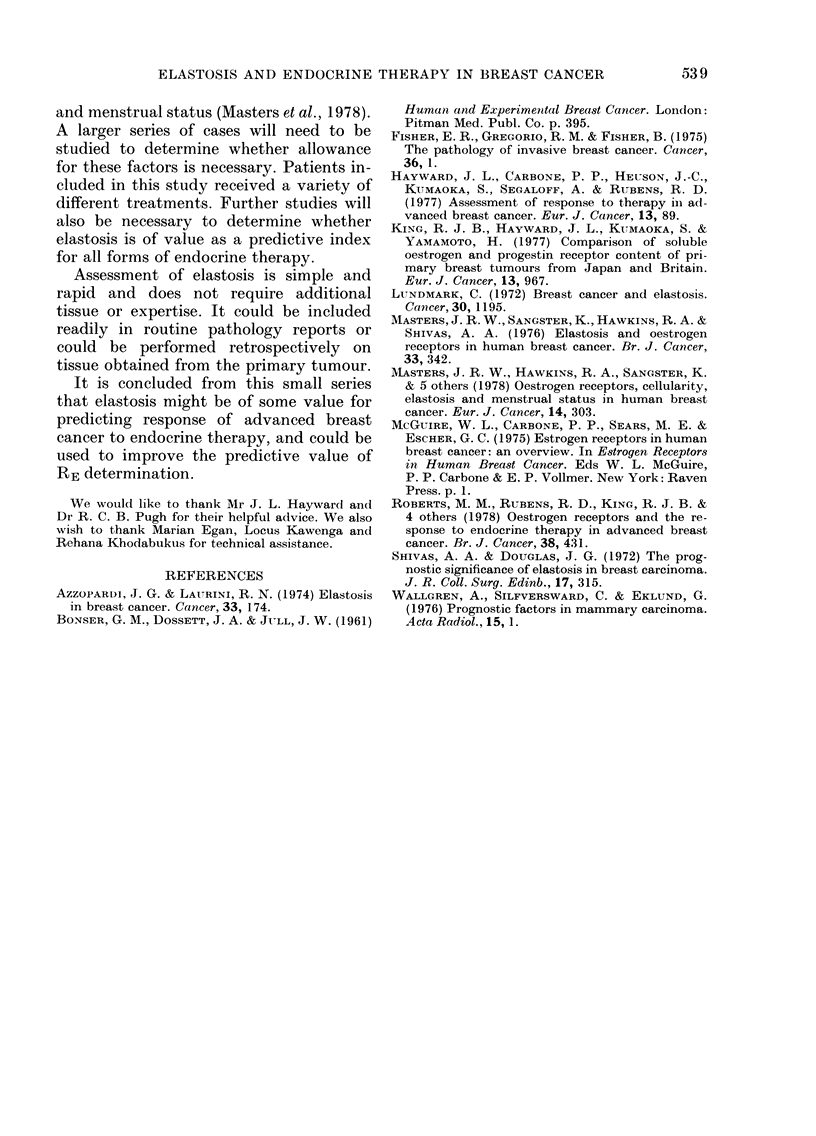# Elastosis and response to endocrine therapy in human breast cancer.

**DOI:** 10.1038/bjc.1979.98

**Published:** 1979-05

**Authors:** J. R. Masters, R. R. Millis, R. J. King, R. D. Rubens

## Abstract

Response to endocrine therapy in 51 patients with advanced breast cancer was compared with the amount of elastosis in histological sections from their primary tumours. There appeared to be an association between elastosis and response: tumours with no elastosis showed a lower rate of response than those with gross elastosis, indicating that this simple method might provide a useful predictive index for response to endocrine therapy. In addition, tumours with oestrogen-receptor activity (a feature associated with a high rate of response) but with no elastosis were unlikely to respond, suggesting that a combination of the 2 predictive indices might be more valuable than either taken alone.


*Br. J. Cancer* (1979), **39**, 536

### ELASTOSIS AND RESPONSE TO ENDOCRINE THERAPY IN HUMAN BREAST CANCER

J. R. W. MASTERS,* R. R. MILLIS,† R. J. B. KING‡ AND R. D. RUBENS†

*From *the Imperial Cancer Research Fund Breast Cancer Unit, Guy's Hospital, London, †the Department of Pathology, Institute of Urology, St Paul's Hospital, London, and ‡the Hormone Biochemistry Department, Imperial Cancer Research Fund, Lincoln's Inn Fields, London*

Received 12 November 1978 Accepted 2 February 1979

**Summary.—Response to endocrine therapy in 51 patients with advanced breast cancer was compared with the amount of elastosis in histological sections from their primary tumours. There appeared to be an association between elastosis and response: tumours with no elastosis showed a lower rate of response than those with gross elastosis, indicating that this simple method might provide a useful predictive index for response to endocrine therapy. In addition, tumours with oestrogen-receptor activity (a feature associated with a high rate of response) but with no elastosis were unlikely to respond, suggesting that a combination of the 2 predictive indices might be more valuable than either taken alone.**

CURRENT METHODS for the selection of patients with advanced breast cancer for endocrine therapy are limited, because none provides absolute means of determining which patients will respond. Although the absence of oestrogen receptor activity ($R_E$) in a tumour correlates well with lack of response to endocrine therapy, not all patients with tumours containing $R_E$ will respond (McGuire *et al.*, 1975; Roberts *et al.*, 1978). Recent studies have indicated that a histological feature of breast cancer, the amount of elastosis in the primary tumour, is associated with menstrual status (Lundmark, 1972; Masters *et al.*, 1978), prognosis (Shivas & Douglas, 1972; Wallgren *et al.*, 1976) and $R_E$ (Masters *et al.*, 1976). These findings suggest that elastosis is related to endocrine status and therefore might be of value as a predictive index for response to endocrine therapy. The present study tests this hypothesis by relating elastosis and response of advanced breast cancer to endocrine therapy.

### MATERIALS AND METHODS

The amount of elastosis was measured qualitatively in histological sections from 51 primary breast cancers from patients who subsequently received endocrine therapy for metastatic disease. Histological sections were stained with orcein to demonstrate elastosis. This stain was compared with the modified Gomori aldehyde-fuchsin stain previously used (Shivas & Douglas, 1972; Masters *et al.*, 1976, 1978) on a series of 30 tumours, and identical results were obtained by 2 observers. The amount of elastosis was categorized as follows: 0 indicated that elastosis was not demonstrable, 1 that it was present to a small or moderate degree, and 2 that there was a gross degree of elastosis. The assessments correspond to those of Shivas & Douglas (1972), in that Category 0 corresponds to Elastica Index 0, Category 1 to Indices + and ++ and Category 2 to Index +++. Focal deposits of elastica around malignant cells and ducts lined by malignant cells, but not the material around blood vessels and ducts lined by non-neoplastic epithelium, formed the basis of the assessment. Diffuse elastosis consisting of indi-

Requests for reprints to: R. R. Millis, Hedley Atkins Unit, New Cross Hospital, Avonley Road, London, SE14 5ER.

vidual coarse and/or fine fibrils distributed in an apparently random array was not included in the assessment. In the current study the amount of elastosis was assessed independently by 2 observers and, in the small number of cases in which disagreement occurred, the slides were re-read and classified by joint decision. All assessments were made without knowledge of the patient's history, treatment or response.

$R_E$ was measured in either the primary or metastatic tumour in 50 of these cases using the method of King *et al.* (1977). Tumours were categorized as $R_E^+$ ($\geqslant$5 fmol protein/ml, detectable) or $R_E^-$ (<5 fmol protein/ml, not detectable).

The endocrine treatment given to patients included oophorectomy (8 patients), oestrogens (12 patients), androgens (11 patients), tamoxifen (13 patients) and hypophysectomy (7 patients). Response was assessed using the UICC criteria (Hayward *et al.*, 1977) which define 4 categories, summarized as follows:

(1) Complete response (CR)—disappearance of all known disease.

(2) Partial response (PR)—50% or more decrease in the sum of the perpendicular axes of measurable lesions. No new lesions. It is not necessary for every lesion to have regressed, but no lesion should have progressed.

(3) No change (NC)—lesions unchanged.

(4) Progressive disease (PD). (a) Mixed—some lesions regress while other progress or new lesions appear. (b) Failure—progression of some or all lesions and/or appearance of new lesions; no lesions regress.

### RESULTS

The results are summarized in Tables I and II. There appears to be an association between elastosis and response to endocrine therapy (see Table I and Fig.). Of the patients with tumours containing Category 0 elastosis, 86% failed to show either complete or partial response to treatment, compared with 74% and 50% with Categories 1 and 2 respectively. This association is even more apparent when the 6 patients achieving a complete response are considered as a separate group. While only 6 of the 51 tumours studied

TABLE I.—*The relationship between the amount of elastosis (categorized as* 0, 1 *or* 2) *and the response (assessed by UICC criteria) of advanced breast cancer to endocrine therapy*

| | Elastosis category | | |
|---|---|---|---|
| Response to therapy | 0 | 1 | 2 |
| Objective regression | 2 | 8 | 3 |
| Complete response | 0 | 3 | 3 |
| Partial response | 2 | 5 | 0 |
| No change | 3 | 5 | 1 |
| Progressive disease | 9 | 18 | 2 |
| Total | 14 | 31 | 6 |

FIG.—The relationship between the amount of elastosis (categorized as 0, 1 or 2) and the response (assessed by UICC criteria) to endocrine therapy of 51 patients with advanced breast cancer.

TABLE II.—*Combined $R_E$ (categorized as + or —) and elastosis (categorized as 0, 1 or 2) as a predictive index for response (assessed by UICC criteria) of advanced breast cancer to endocrine therapy*

| $R_E$ | Elastosis | Responders (PR+CR) |
|---|---|---|
| + | 0 | 1/8 (13%) |
| | 1 | 8/19 (42%) |
| | 2 | 3/5 (60%) |
| — | 0 | 1/6 (17%) |
| | 1 | 1/11 (9%) |
| | 2 | 0/1 (0%) |

showed Category 2 elastosis, 3 of these achieved a complete response. This finding is statistically highly significant ($P < 0{\cdot}005$).

The result of combining elastosis assessment with $R_E$ to produce a single predictive index for response is shown in Table II. Of the $R_E^+$ tumours, only 13% with Category 0 elastosis responded to treatment, compared with 42% and 60% respectively with Categories 1 and 2.

### DISCUSSION

The results indicate that the assessment of elastosis in primary breast cancers might be used in the selection of patients receiving endocrine therapy for metastatic disease (Table I). First, in relation to patients who will not respond to endocrine therapy, in this small series only 2/14 patients (14%) with Category 0 elastosis in their tumours showed any objective response. Second, with respect to the patients who will respond to endocrine therapy, 3/6 patients (50%) with Category 2 elastosis achieved a complete response.

A combination of $R_E$ and elastosis assessments provides a better predictive index for response to endocrine therapy than either feature taken alone. The results indicate that $R_E^+$ tumours with Category 0 elastosis are unlikely to respond to therapy (Table II).

The importance of using objective criteria to assess tumour response is well recognized. This preliminary study is based on data from 51 patients. Although more patients were initially included in this series, a large number had to be excluded either because the primary tumour was not available or because objective assessment using UICC criteria (Hayward *et al.*, 1977) was not possible. It is hoped that this preliminary communication will encourage further investigation of elastosis as a predictive index for the response of advanced breast cancer to endocrine therapy.

Several authors have discussed methods of grading elastosis (Lundmark, 1972; Shivas & Douglas, 1972; Azzopardi & Laurini, 1974), but these assessments are not comparable because of differences in the sites of elastica included in the grading. For example, in contrast to the present study, Azzopardi & Laurini (1974) included increased elastosis around ducts lined by non-neoplastic epithelium and vasculature in their assessment. Further studies will be necessary to determine whether such elastosis is of significance in relation to response to endocrine therapy.

The reported incidence of elastosis in breast tumours varies from 45 to 88% according to the type of tumour examined and the method of assessment (Bonser *et al.*, 1961; Lundmark, 1972; Azzopardi & Laurini, 1974; Fisher *et al.*, 1975). Elastosis is uncommon in mucoid and medullary carcinomas (Azzopardi & Laurini, 1974). However, there is no evidence that these relatively uncommon histological types of tumour are less likely to respond to endocrine therapy. Thus the absence of elastosis in certain histological types of carcinoma may not be of predictive significance.

Previous studies have shown a positive association between elastosis and $R_E$ (Masters *et al.*, 1976, 1978). In the present study a similar trend was found. Of the tumours with Category 0 elastosis, 8/14 (57%) contained $R_E$, compared with 19/30 (63%) and 5/6 (83%) of tumours with Categories 1 and 2 elastosis respectively.

The amount of elastosis in breast cancers is related to age (Lundmark, 1972)

and menstrual status (Masters *et al.*, 1978). A larger series of cases will need to be studied to determine whether allowance for these factors is necessary. Patients included in this study received a variety of different treatments. Further studies will also be necessary to determine whether elastosis is of value as a predictive index for all forms of endocrine therapy.

Assessment of elastosis is simple and rapid and does not require additional tissue or expertise. It could be included readily in routine pathology reports or could be performed retrospectively on tissue obtained from the primary tumour.

It is concluded from this small series that elastosis might be of some value for predicting response of advanced breast cancer to endocrine therapy, and could be used to improve the predictive value of $R_E$ determination.

We would like to thank Mr J. L. Hayward and Dr R. C. B. Pugh for their helpful advice. We also wish to thank Marian Egan, Locus Kawenga and Rehana Khodabukus for technical assistance.

### REFERENCES

AZZOPARDI, J. G. & LAURINI, R. N. (1974) Elastosis in breast cancer. *Cancer*, **33**, 174.

BONSER, G. M., DOSSETT, J. A. & JULL, J. W. (1961) *Human and Experimental Breast Cancer*. London: Pitman Med. Publ. Co. p. 395.

FISHER, E. R., GREGORIO, R. M. & FISHER, B. (1975) The pathology of invasive breast cancer. *Cancer*, **36**, 1.

HAYWARD, J. L., CARBONE, P. P., HEUSON, J.-C., KUMAOKA, S., SEGALOFF, A. & RUBENS, R. D. (1977) Assessment of response to therapy in advanced breast cancer. *Eur. J. Cancer*, **13**, 89.

KING, R. J. B., HAYWARD, J. L., KUMAOKA, S. & YAMAMOTO, H. (1977) Comparison of soluble oestrogen and progestin receptor content of primary breast tumours from Japan and Britain. *Eur. J. Cancer*, **13**, 967.

LUNDMARK, C. (1972) Breast cancer and elastosis. *Cancer*, **30**, 1195.

MASTERS, J. R. W., SANGSTER, K., HAWKINS, R. A. & SHIVAS, A. A. (1976) Elastosis and oestrogen receptors in human breast cancer. *Br. J. Cancer*, **33**, 342.

MASTERS, J. R. W., HAWKINS, R. A., SANGSTER, K. & 5 others (1978) Oestrogen receptors, cellularity, elastosis and menstrual status in human breast cancer. *Eur. J. Cancer*, **14**, 303.

MCGUIRE, W. L., CARBONE, P. P., SEARS, M. E. & ESCHER, G. C. (1975) Estrogen receptors in human breast cancer: an overview. In *Estrogen Receptors in Human Breast Cancer*. Eds W. L. McGuire, P. P. Carbone & E. P. Vollmer. New York: Raven Press. p. 1.

ROBERTS, M. M., RUBENS, R. D., KING, R. J. B. & 4 others (1978) Oestrogen receptors and the response to endocrine therapy in advanced breast cancer. *Br. J. Cancer*, **38**, 431.

SHIVAS, A. A. & DOUGLAS, J. G. (1972) The prognostic significance of elastosis in breast carcinoma. *J. R. Coll. Surg. Edinb.*, **17**, 315.

WALLGREN, A., SILFVERSWARD, C. & EKLUND, G. (1976) Prognostic factors in mammary carcinoma. *Acta Radiol.*, **15**, 1.